# A systematic evaluation of hybridization-based mouse exome capture system

**DOI:** 10.1186/1471-2164-14-492

**Published:** 2013-07-21

**Authors:** Qingsong Gao, Wei Sun, Xintian You, Sebastian Froehler, Wei Chen

**Affiliations:** 1 Laboratory for Novel Sequencing Technology, Functional and Medical Genomics, Berlin Institute for Medical Systems Biology, Max-Delbrück-Centrum für Molekulare Medizin, Robert-Rössle-Straße 10, Berlin 13125, Germany

**Keywords:** Mouse exome capture system, Sequence divergence, Capture bias, Efficiency of variant detection

## Abstract

**Background:**

Exome sequencing is increasingly used to search for phenotypically-relevant sequence variants in the mouse genome. All of the current hybridization-based mouse exome capture systems are designed based on the genome reference sequences of the C57BL/6 J strain. Given that the substantial sequence divergence exists between C57BL/6 J and other distantly-related strains, the impact of sequence divergence on the efficiency of such capture systems needs to be systematically evaluated before they can be widely applied to the study of those strains.

**Results:**

Using the Agilent SureSelect mouse exome capture system, we performed exome sequencing on F1 generation hybrid mice that were derived by crossing two divergent strains, C57BL/6 J and SPRET/EiJ. Our results showed that the C57BL/6 J-based probes captured the sequences derived from C57BL/6 J alleles more efficiently and that the bias was higher for the target regions with greater sequence divergence. At low sequencing depths, the bias also affected the efficiency of variant detection. However, the effects became negligible when sufficient sequencing depth was achieved.

**Conclusion:**

Sufficient sequence depth needs to be planned to match the sequence divergence between C57BL/6 J and the strain to be studied, when the C57BL/6 J–based Agilent SureSelect exome capture system is to be used.

## Background

Massive parallel sequencing has revolutionized the search for sequence variants in the human genome as well as other model organisms. Compared with the Sanger method, these so-called next-generation sequencing platforms can sequence DNA much faster and at a much lower cost [1,2]. The resequencing of an entire human genome can now be achieved within days at the cost of a few thousand dollars. In spite of such dramatically improved performance, the current technology does not allow routine screening of the complete genome for a large number of samples in an economically-efficient manner [3]. Even when the cost of whole genome sequencing breaks the much-anticipated $1000 threshold, the necessary computational workload could still remain burdensome and limit its widespread implementation outside major genome centres. In comparison, targeted sequencing is less expensive and generates datasets several orders of magnitude smaller [4-8]. In recent years, exome sequencing (i.e. the sequencing of the full complement of protein-coding exons in the genome) has therefore become a favoured approach in identifying sequence variants causing rare as well as common human diseases [9-11].

Over the last few decades, the mouse has emerged as a preeminent model organism for exploring human biology. Since over 90% of known mouse genes have an orthologue in the human genome, identification of genetic defects responsible for certain phenotypes in mice can directly indicate the genes involved in human diseases. Recently, given the success of exome sequencing in identifying human disease-causing variants, hybridization-based exome capture systems have been developed for mouse and are now commercially available [12,13]. The current design of mouse exome capture probes is based on the genome reference sequences of the C57BL/6 J strain and the robustness of applying such systems in other strains has been shown in a study that mapped putative *N*-ethyl-*N*-nitrosourea (ENU)-induced mutations in four different inbred *Mus musculus* strains [12]. However, the marginal differences between the genomes of C57BL/6 J and the other strains used in that study do not allow for a systematic evaluation of the effects of sequence divergence on the efficiency of sequence capture and variant detection. In addition, the effect of sequence divergence could be even greater in a mixed genetic background.

The genome of the SPRET/EiJ strain has recently been sequenced. Compared with that of C57BL/6 J, the genome of SPRET/EiJ contains about 35.4 million single nucleotide variants (SNVs) and 4.5 million insertion and deletions (indels) [14]. In this study, to gain a better understanding of how sequence divergence could affect the efficiency of the current exome capture system, especially in a mixed genetic background, we performed exome sequencing in a F1 hybrid mouse generated by crossing the two strains with a high degree of sequence variation. After mapping the sequencing reads separately to the genomes of the two parental strains, we observed that probes captured the sequences derived from C57BL/6 J alleles more efficiently and the bias was higher for target regions with greater sequence divergence. Such bias also reduced the efficiency of variant detection which could be counteracted by higher sequencing depth.

## Results and discussion

### Exome capture

**Figure 1:**
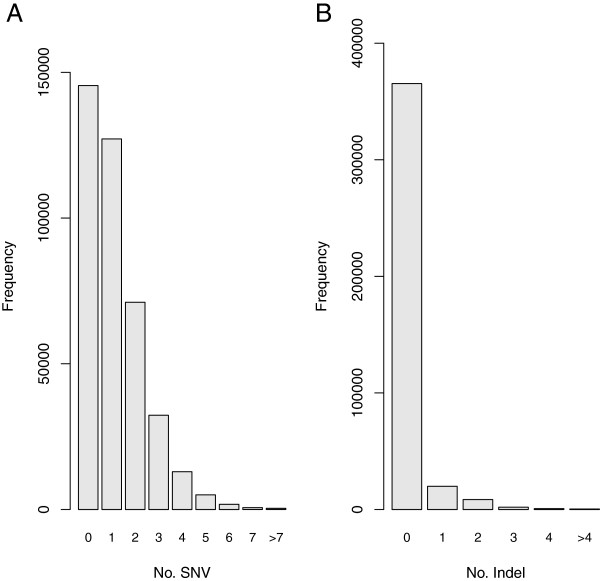
**Histograms of the targets with different levels of sequence divergence.** The 396,870 targets that were used for evaluating capture efficiency were grouped based on the number of SNVs **(A)** or indels **(B)** between the C57BL/6 J and SPRET/EiJ genomes. The numbers of targets (Y axis) with different numbers of SNVs (X axis) or indels (X axis) were shown.

To evaluate whether sequence divergence could affect exome capture, especially in a mixed genetic background, we performed exome sequencing on a F1 hybrid mouse derived from crossing C57BL/6 J and SPRET/EiJ mice using an Agilent SureSelect XT Mouse All Exon Kit (Methods). With a design based on the genome sequences of C57BL/6 J strain (UCSC genome browser mm9), the kit contains 565,918 probes of length 120 nucleotides (nt) that are targeted at the exonic regions of 24,306 genes. Among all the target regions, we could unambiguously identify orthologous regions in the SPRET/EiJ genome for 404,992 targets (Methods). To control for complicating factors beyond exome capture bias, we simulated three exome sequencing datasets without any allelic bias in capture efficiency (Methods). Furthermore, we performed whole genome sequencing (WGS) on the same F1 hybrid mouse. Only the 396,870 targets that did not show significant allelic bias in sequencing depth from either simulation or WGS were retained for further analysis (Methods, Additional files 1 and 2). Among these targets the sequences of 136,956 targets are identical between the two strains, whereas the remaining 259,914 targets contain up to 15 SNVs and 10 indels (Figure 1).

**Figure 2:**
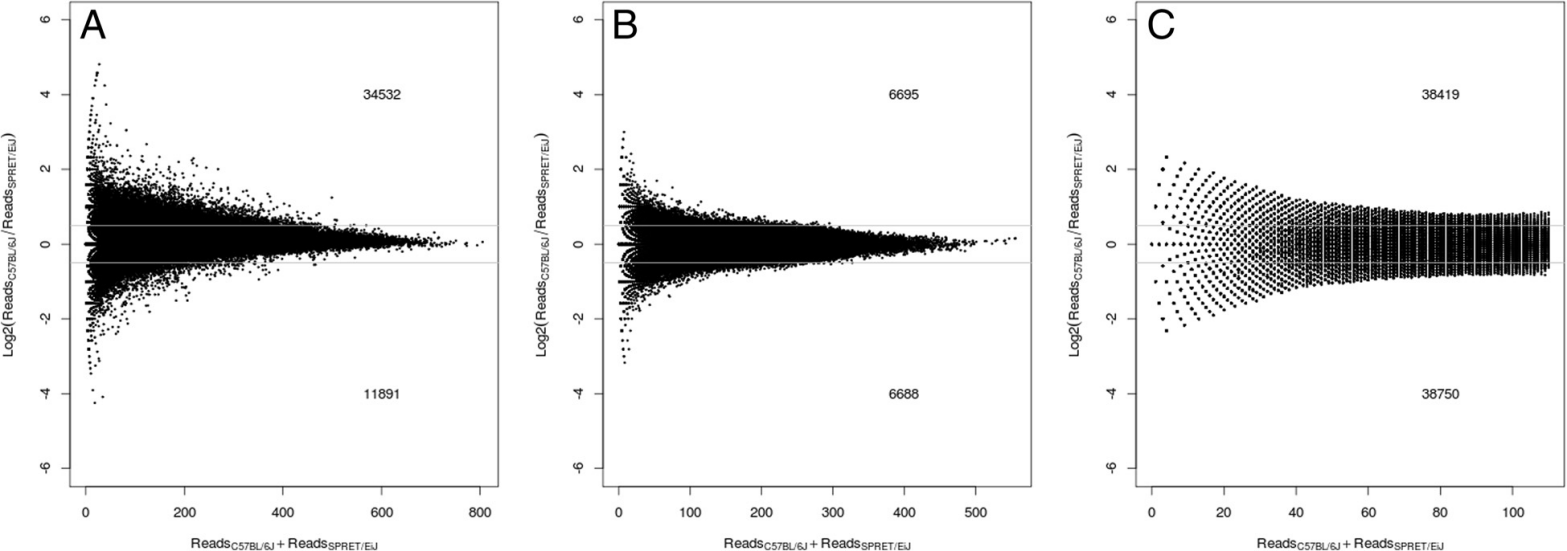
**Evaluation of allelic bias in capture efficiency.** MA plots comparing the number of sequencing reads that overlapped with a target derived from C57BL/6 J with that from SPRET/EiJ allele based on exome sequencing **(A)**, simulated data **(B)** or WGS **(C)**. The sum of the number of sequencing reads that could be mapped only to either genome was plotted on the X axis, and the log2 transformed ratio between the number of reads derived from C57BL/6 J and that from SPRET/EiJ allele was on the Y axis. The numbers of targets with log2 transformed ratio > =0.5 or < = −0.5 were shown in the respective figure. In exome sequencing, the reads mapped only to C57BL/J were clearly overrepresented whereas such bias did not exist in the simulated or WGS data.

In total, we performed three replicate exome capture experiments, each of which being then sequenced for single-end 101 nt on one single lane of the Illumina HiSeq 2000 flowcell. An average of 196.9 million reads was generated from each replicate, among which 85.5% could be mapped to the genome of either strain (Table 1). Between 51.8 and 53.4 million non-redundant reads mapped to the 396,870 target regions were used to assess the allelic bias in capture efficiency (Table 1). More specifically, for each of these targets, we calculated the number of reads that could be mapped only to the C57BL/6 J or SPRET/EiJ genome. As shown in Table 1 and Figure 2A, the reads mapped only to C57BL/J alleles were clearly overrepresented. The fact that such bias did not exist in the simulation or WGS data indicated the probes captured the target sequences derived from C57BL/J allele more efficiently (Table 1 and Figure 2B, C).

**Table 1:** Summary of mapping results from exome sequencing, WGS and simulated exome sequencing

	**Exome 1 (million)**	**Exome 2 (million)**	**Exome 3 (million)**	**WGS (million)**	**Simulation1 (million)**	**Simulation2 (million)**	**Simulation3 (million)**
No. of total reads	201.4	195.1	194.2	1097.0	75.0	75.0	75.0
No. of reads uniquely mapped to either genome	171.9	167.0	166.1	921.1	74.9	74.9	74.9
No. of non-redundant reads mapped to the targets on either genome	53.4	52.4	51.8	21.3	58.8	58.8	58.8
No. of non-redundant reads mapped only to the targets from C57BL/6 J	19.0	18.7	18.5	7.1	20.0	20.0	20.0
No. of non-redundant reads mapped only to the targets from SPRET/EiJ	17.4	17.0	16.9	7.1	20.0	20.0	20.0

**Figure 3:**
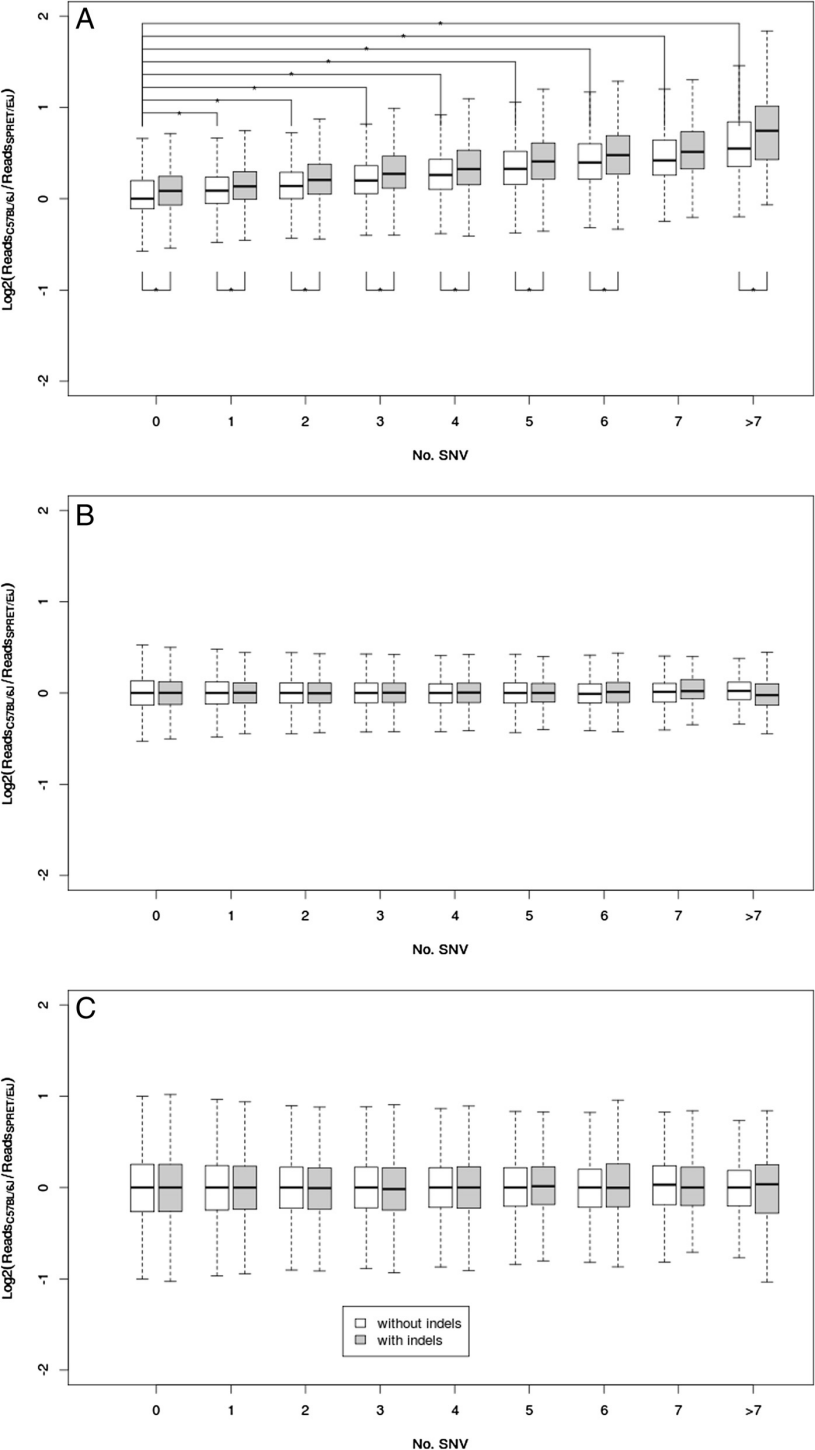
**Effects of sequence divergence on capture bias.** Boxplots showed the distribution of capture bias (i.e. log2 transformed ratio between the number of reads derived from C57BL/6 J and that from SPRET/EiJ allele) for the targets without indels grouped by the number of SNVs (white boxes) based on exome sequencing **(A)**, simulated exome sequencing data **(B)** or WGS **(C)**. In exome sequencing, compared with the targets with identical sequence between the two genomes, the targets containing SNVs showed significantly higher capture biases toward C57BL/J. The more SNVs the targets contained, the higher the bias was observed. Compared with the targets containing no indels (white boxes), those containing the same number of SNVs, but also indels (grey boxes), showed significantly higher capture biases toward C57BL/J. Both biases were not observed in simulated and WGS data. * denotes statistically significant differences (P < 0.01).

Assuming that the allelic capture bias observed above was largely due to the sequence divergence between the two strains, we assessed the impact of sequence divergence in greater detail. Here we checked the effect of SNVs and indels, separately. In total, 365,369 targets containing no indels between the two strains were grouped based on the number of SNVs. As shown in Figure 3A, compared with those targets with identical sequences, the targets containing SNVs showed significant capture biases toward C57BL/J. The more SNVs the targets contained, the higher the bias was observed (Figure 3A). Interestingly, the distribution of multiple SNVs within the target regions also had an impact on the capture bias (Additional file 3). Furthermore, a clear effect on allelic bias could also be seen for indels by comparing the targets containing indels to those containing the same number of SNVs, but no indels (Figure 3A). Notably, such biases did not exist in simulation or WGS data (Figure 3B, C).

### Variant detection

**Figure 4:**
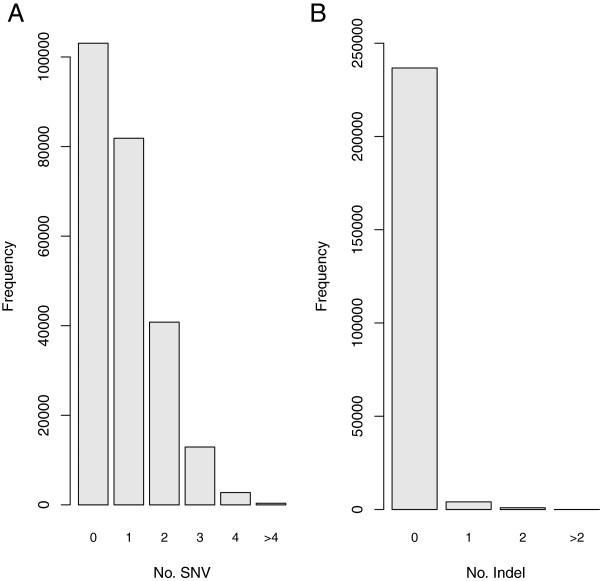
**Histograms of the targets with different sequence divergence.** The 241,729 targets without mapping biases that were used for evaluating the efficiency of variant detection were grouped based on the number of SNVs **(A)** or indels **(B)** between the C57BL/6 J and SPRET/EiJ genomes. The numbers of targets (Y axis) with different numbers of SNVs (X axis) or indels (X axis) were shown.

We next sought to check whether the capture bias would affect the efficiency of variant detection. For this purpose, we mapped the exome sequencing reads only to the C57BL/6 J genome and aimed to identify the heterozygous variants derived from the SPRET/EiJ allele. Using our mapping procedure, the sequencing reads derived from SPRET/EiJ allele containing >5 SNVs and indels could not be mapped on the C57BL/6 J genome. Since we focused on the effect of capture bias, we first needed to filter out the target regions that could be potentially affected by such mapping bias. To identify these specific regions, we mapped the simulated exome sequencing reads to either both genomes together or to only the C57BL/6 J genome. The numbers of sequencing reads that could be mapped using these two strategies were then compared for each of the 396,870 targets. Mapping bias was indicated if more reads could be mapped based on the ‘two genome approach’. To exclude the effect of mapping bias, we retained only the 241,729 targets on which the number of reads mapped using the two mapping strategies was the same in all the three simulated datasets. In total, there were 192,614 SNVs and 5511 indels falling within these targets and the distribution of the number of SNVs and indels across these targets were shown in Figure 4.

**Figure 5:**
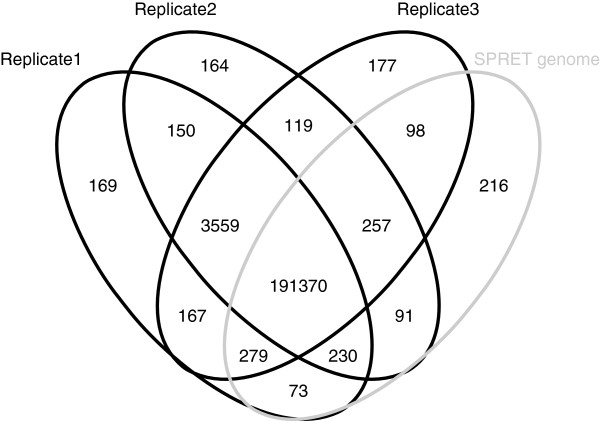
**Number of SNVs detected by the three exome sequencing replicate experiments.** In total, 194,929 SNVs were identified in all the three replicates, whereas 1202 and 772 were found in two and one replicates, respectively. Of the 192,614 SNVs present in the SPRET/EiJ genome reference sequences, 99.4%, 99.8% and 99.9% could be identified in at least three, two and one replicates, respectively. A total of 4505 SNVs identified in at least one replicates were not found in the SPRET/EiJ genome reference sequences. 3559 and 436 of them could be detected in three and two replicates, respectively.

We called variants within these targets based on the three exome sequencing datasets (Methods). Given the relatively limited performance of current variant callers on the indel identification, we focused our analysis on SNVs. As shown in Figure 5, 194,929 SNVs were identified in all the three replicates, whereas 1202 and 772 were only found in two and one replicates, respectively. Of the 192,614 SNVs present in the SPRET/EiJ genome reference sequences, 99.4%, 99.8% and 99.9% could be identified in at least three, two and one replicates, respectively. A total of 4505 SNVs identified in at least one replicates were not found in the SPRET/EiJ genome reference sequences. These variants could represent false positive findings. However, 3559 and 436 of them could be detected in three and two replicates, respectively, implicating that they might be true variants but absent in the reference sequences. Indeed, 3092 were annotated as mouse SNPs in Sanger Database (http://www.sanger.ac.uk/resources/mouse/genomes/) [14] and 3618 could also be detected using our WGS data.

**Figure 6:**
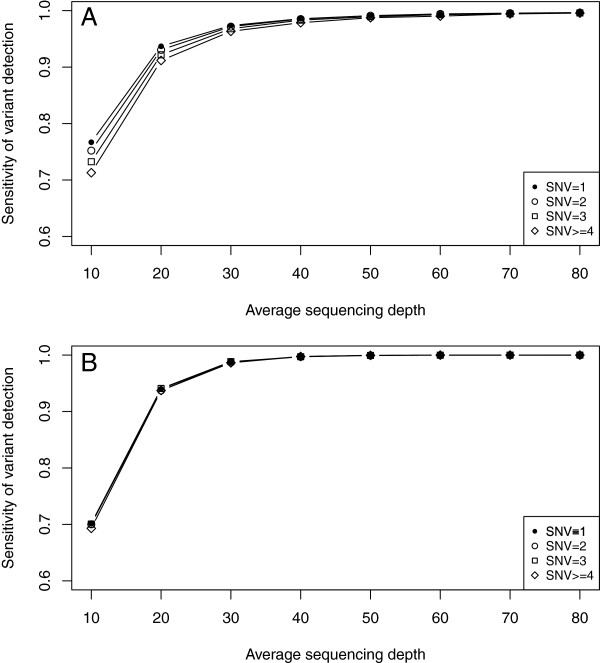
**The sensitivity of variant detection at lower sequencing depths for the targets containing different SNVs.** The sensitivity of variant detection (Y axis) was plotted for the targets containing different number of SNVs (different lines) at different average sequencing depths (X axis) based on exome sequencing **(A)** or simulated data **(B)**. In exome sequencing, the sensitivity of variant detection became decreased with the reduced sequencing depths. At lower sequencing depths, the sensitivity was lower for the targets containing more SNVs. For example, at average depth of 10, whereas a sensitivity of 76.7% was achieved for the targets containing only one SNV, the sensitivity for the targets with four SNVs dropped to 71.2%. Such a trend was not observed for simulated data, implicating the decreased sensitivity was largely due to capture bias.

We achieved an average sequencing depth of 91.1 for all nucleotide positions within the 241,729 targets using one lane of a single flowcell. 98% and 100% of all the positions were covered by more than 20 and 5 sequencing reads, respectively. Of note, such a sequencing depth was sufficiently high to discover most (99.7%) of the variants even though the exome capture was biased against variant carrying alleles. Therefore, we then addressed how the variant detection would work at lower sequencing depth and whether the exome capture bias could then have an effect on variant detection. We randomly sampled a subset of reads from the first exome sequencing replicate to cover the targets at an average sequencing depth of 10, 20, 30, 40, 50, 60, 70 and 80, respectively. As expected, the sensitivity decreased with the reduced sequencing depth. We then grouped the targets based on the number of SNVs they contained. As shown in Figure 6A, at a lower sequencing depth, the sensitivity was lower for the targets containing more SNVs. For example, at average depth of 10, whereas a sensitivity of 76.7% was achieved for the targets containing only one SNV, the sensitivity for the targets with four SNVs dropped to 71.2%. A similar trend was not observed for simulated data, implicating the decreased sensitivity was largely due to capture bias (Figure 6B).

In this study, on one hand, to avoid the effect of mapping bias, we focused our analysis only on the targets containing no more than 6 SNVs and 4 indels. As shown in the previous section, the greater the sequence divergence, the higher capture bias was observed. Therefore it is conceivable that the efficiency in variant detection could be even lower for the targets with higher sequencing divergence. On the other hand, we used the capture system to detect heterozygous variants in a F1 hybrid mouse. For many research projects that work on pure inbred strains and/or only search for homozygous variants, the effect of sequence divergence might be more subtle than what has been demonstrated here.

## Conclusions

With the enormous progress in the field of human genetics, exome capture systems have been developed to search for phenotypically-relevant mutations in mice. Unlike the human genome in which sequence differences among individuals are rather limited, the sequence divergence between different mouse strains could be substantial. However, it had not been extensively explored whether the sequence divergence could affect the efficiency of hybridization-based capture systems designed based on a reference genome (C57BL/6 J) when applied in the study of distantly-related strains. In this study, we performed an exome capture and sequencing on a F1 hybrid mouse generated by crossing the C57BL/6 J and SPRET/EiJ strains. Our results clearly demonstrated that the probes captured the sequences derived from C57BL/6 J alleles more efficiently and the bias increased for the target regions with higher sequence divergence. This bias also affected the efficiency of variant detection. The effect, however, could be counteracted by increasing sequencing depth. For example, to achieve a 99% detection sensitivity, an average sequencing depth of 50 or 60 would be required for the regions containing one or four SNVs. Therefore, in the design of exome sequencing in different mouse strain backgrounds, sufficient sequence depths need to be planned to match the sequence divergence between the strain and C57BL/6 J.

## Methods

### Exome capture and sequencing

Whole exome sequencing libraries were prepared using an Agilent SureSelect XT Mouse All exon kit. Genomic DNA samples extracted from mouse (a female F1 hybrid mouse derived from crossing C57BL/6 J and SPRET/EiJ) liver were used to generate Illumina Paired-End precapture libraries according to the manufacturer’s protocol (Agilent). After determining concentration and quality, 3 μg genomic DNA was sheared into fragments with an average length between 150 and 200 bp using a Covaris S2 system (Covaris). The fragmented DNA was end-repaired in 100 μl total reaction volume containing 48 μl sheared DNA, 10 μl 10X buffer, 1.6 μl dNTP, 1 μl T4 DNA polymerase, 2 μl Klenow DNA polymerase and 2.2 μl T4 Polynucleotide Kinase at 20°C for 30 minutes. A-tailing was performed in a total reaction volume of 50 μl containing end-repaired DNA, 5 μl 10X buffer, 3 μl Klenow Fragment, 1 μl dATP and 11 μl water at 37°C for 30 minutes. Illumina adapter ligation was performed in a total reaction volume of 50 μl containing 10 μl 5X buffer, 10 μl ligase and 10 μl adaptor oligo mix at room temperature for 30 minutes. After ligation, PCR with Illumina PE 1.0 and SureSelect GA Indexing Pre Capture PCR Reverse Primer was performed in 50 μl reactions containing 10 μl 5x Herculase II Rxn Buffer, adaptor-ligated DNA, 1.25 μl of each primer, 0.5 μl 100 mM dNTP mix and 1 μl Herculase II Fusion DNA Polymerase. The standard thermocycling for PCR was 2 minutes at 98°C for the initial denaturation followed by 6 cycles of 30 seconds at 98°C, 30 seconds at 65°C and 60 seconds at 72°C and a final extension for 10 minutes at 72°C. Agencourt® XP® Beads (Beckman Coulter Genomics) was used to purify DNA after each enzymatic reaction. After bead purification, the amount and size of PCR products were determined using an Agilent 2100 Bioanalyzer (Agilent) and Qubit (Life Technology).

Then, 500 ng of Illumina paired-end pre-capture library DNA was hybridized to Agilent SureSelect mouse exome capture probes according to the manufacturer’s specifications. After assessing the quality of capture libraries using Agilent 2100 Bioanalyzer, the captured library was sequenced using Illumina HiSeq 2000 system according to the manufacturer’s protocol. Each library was sequenced in a separate lane in a 101 nt single-end sequencing format.

### Whole genome sequencing (WGS)

An Illumina paired-end sequencing library was generated from the same genomic DNA used for exome sequencing (see above) according to the manufacturer’s protocol (Illumina). The procedure is the same as the one for constructing the precapture library described above. The library was sequenced in seven lanes in a 2 x 100 nt paired-end sequencing format using Illumina HiSeq 2000 system according to manufacturer’s protocol. In this study, to be comparable with the format of exome sequencing, we analyzed only the first reads from WGS.

### Mapping orthologous target regions in the SPRET/EiJ genome

The Agilent SureSelect XT Mouse All Exon Kit was designed based on the C57BL/6 J genome (UCSC mm9). The BED file containing all the target regions on the C57BL/6 J genome was downloaded from the Agilent website (http://www.genomics.agilent.com). The C57BL/6 J sequences of all the targets except those from chrY and mitochondrial were downloaded from UCSC genome browser (mm9). The SPRET/EiJ genome sequences were downloaded from the Sanger Institute (http://www.sanger.ac.uk/resources/mouse/genomes/). The C57BL/6 J sequences were then aligned to the SPRET/EiJ genome using the BLAST tool with default parameters [15]. Only the targets that could be uniquely mapped in the same chromosome as in the C57BL/6 J genome were retained. In addition, we excluded the targets that did not lie in the same 5’-to-3’ order with their neighbouring targets as in the C57BL/6 J genome. Each of the remaining targets was then extended 100 nt from both 5’ and 3’ ends. Those targets that were extended into the regions without reference sequences (shown as ‘N’s in the genome reference sequences) in either genome were discarded.

### Simulation of exome sequencing

We simulated the exome sequencing datasets by randomly extracting 75 million single-end 101nt sequencing reads from the 404,992 target regions on both the C57BL/6 J and SPRET/EiJ genomes. These targets regions were selected to have an unambiguous one-to-one orthologue between the two strains as described above. In total, we repeated the simulation three times and each simulation was performed as following:

1) Randomly select one target *i*. Here the target from the C57BL/6 J and SPRET/EiJ genomes was treated as different ones. The probability of choosing a target *i* was its length divided by the cumulative length of all the 809,984 (2 * 404,992) targets.

2) Randomly select a start position *j* of a sequencing read within the target *i*. Here each of the positions between (*i*_*start*_ − 100) and *i*_*end*_ (*i*_*start*,_ the start position of target *i*; *i*_*end*_, the end position of target *i* ) was chosen with equal probability. The end position of the sequencing read was then *j* + 100.

3) Randomly select the strand. Here forward or reverse strand was chosen with equal probability

4) Extract the sequence of the read from either C57BL/6 J or SPRET/EiJ genome according to 2) and 3). The quality scores for all the bases were set to be 40 (the maximum in the Sanger scale);

5) Repeat step 1 to step 4 for 75 million times to generate 75 million sequencing reads.

### Evaluation of allelic bias in capture efficiency

Raw sequencing reads in FASTQ format from either exome sequencing, WGS, or simulated datasets, were aligned separately to both C57BL/6 J and SPRET/EiJ genomes with the Burrows-Wheeler Aligner (BWA) (Additional file 4) [16]. Reads that could be mapped to multiple positions in one or both genomes were discarded. Reads that could be mapped to both genomes with the same edit distance were also excluded for the analysis of allelic bias in capture efficiency since their allelic origins could not be determined. PCR duplicates were removed with Picard MarkDuplicates (http://picard.sourceforge.net) (Additional file 4). Of the remaining reads, if a read could be mapped only to one genome, the allelic origin is obvious, while if a read could be mapped to both genomes with different edit distances, the strain with smaller edit distance was assigned as the allelic origin. To evaluate allelic bias in capture efficiency, for each of the target, the number of sequencing reads that were overlapped with the target on C57BL/6 J genome was compared to the number of overlapping sequencing reads derived from SPRET/EiJ genome. The sequencing depth of each nucleotide position was designated as the number of sequencing reads overlapped with that position. The sequencing depth of a target region was calculated as the mean depth of all nucleotide positions within the target.

### Variant detection

Raw sequencing reads from exome sequencing, WGS or simulation were aligned to the C57BL/6 J genome using BWA with the same parameters as described in “Evaluation of allelic bias in capture efficiency”. PCR duplicates were removed with Picard MarkDuplicates with the same parameters as described in “Evaluation of allelic bias in capture efficiency”. The Genome Analysis Toolkit (GATK) variant calling pipeline (version 2.2.15) was then used to identity SNVs on each data set separately [17]. In brief, the RealignerTargetCreator and the IndelRealigner tools were used for local realignment around indels, the BaseRecalibrator and the PrintReads tools were used for quality score recalibration, the UnifiedGenotyper tool was used for variant calling, and the VariantRecalibrator and ApplyRecalibration tools were used for variant quality score recalibration. All the commands were listed in Additional file 4.

### Animal ethics statement

Mice were housed and maintained in a temperature-controlled, 12-hour light/dark cycle environment with ad libitum access to regular chow food in accordance to requirements established by Landesamt für Gesundheit und Soziales (Lageso). All experimental procedures were approved under protocol T 0436/08.

## Availability of supporting data

The data sets supporting the results of this article are available in the European Nucleotide Archive (accession number ERP002193).

## Abbreviations

ENU: *N*-ethyl-*N*-nitrosourea; SNV: Single nucleotide variant; indel: Insertion and deletion; WGS: Whole genome sequencing; nt: Nucleotide.

## Competing interests

The authors declare that they have no competing interests.

## Authors’ contributions

WC conceived and designed the project. QG analyzed the data. WS helped with the experiments. XY and SF helped with data analysis. QG and WC wrote the manuscript. All authors read and approved the final manuscript.

## Supplementary Material

Additional file 1 **Exclusion of targets with other complicating factors based on simulated data.** (A-C) MA plots comparing the number of sequencing reads that overlapped with a target derived from C57BL/6 J with that from SPRET/EiJ allele based on three simulated exome sequencing data. The sum of the number of sequencing reads that could be mapped only to either genome was plotted on the X axis, and the log2 transformed ratio between the number of reads derived from C57BL/6 J and that from SPRET/EiJ allele was on the Y axis. The red dots represent outlier targets, i.e. their log2 ratio falling outside the 0.5% ~ 99.5% quantiles. (D) The overlap of outlier targets identified in the three replicate simulations. The 780 targets identified as outliers in at least two replicates (shown in red) were discarded in subsequent analysis. Click here for file

Additional file 2 **Exclusion of targets with other complicating factors based on WGS data.** MA plot comparing the number of sequencing reads that overlapped with a target derived from C57BL/6 J with that from SPRET/EiJ allele based on WGS data. The sum of the number of sequencing reads that could be mapped only to either genome was plotted on the X axis, and the log2 transformed ratio between the number of reads derived from C57BL/6 J and that from SPRET/EiJ allele was on the Y axis. The red dots represent outlier targets, i.e. their log2 ratio falling outside the 0.5% ~ 99.5% quantiles or the sum of the number of sequencing reads being more than 110. All the 7525 outliers were discarded in subsequent analysis. Click here for file

Additional file 3 **Impact of distribution of multiple SNVs in the target regions on the capture bias.** To check whether the SNVs distribution within the target regions could affect the capture efficiency, we focused on the 64,470 targets containing two SNVs, but no indels and grouped them by the distance between the two SNVs. The distance between the two SNVs was shown on the X axis, and the log2 transformed ratio between the number of reads derived from C57BL/6 J and that from SPRET/EiJ allele was on the Y axis. Compared with the targets with two SNVs being far away, those with the two SNVs being right next to each other displayed higher biases in the capture efficiency. * denotes statistically significant differences. Click here for file

Additional file 4 List of data analysis commands. Click here for file
