# Supplementary figures and images for: A systematic evaluation of hybridization-based mouse exome capture system
Source: BMC Genomics. 2013 Jul 21;14:492. doi: 10.1186/1471-2164-14-492 (PMC3722074; doi:10.1186/1471-2164-14-492)

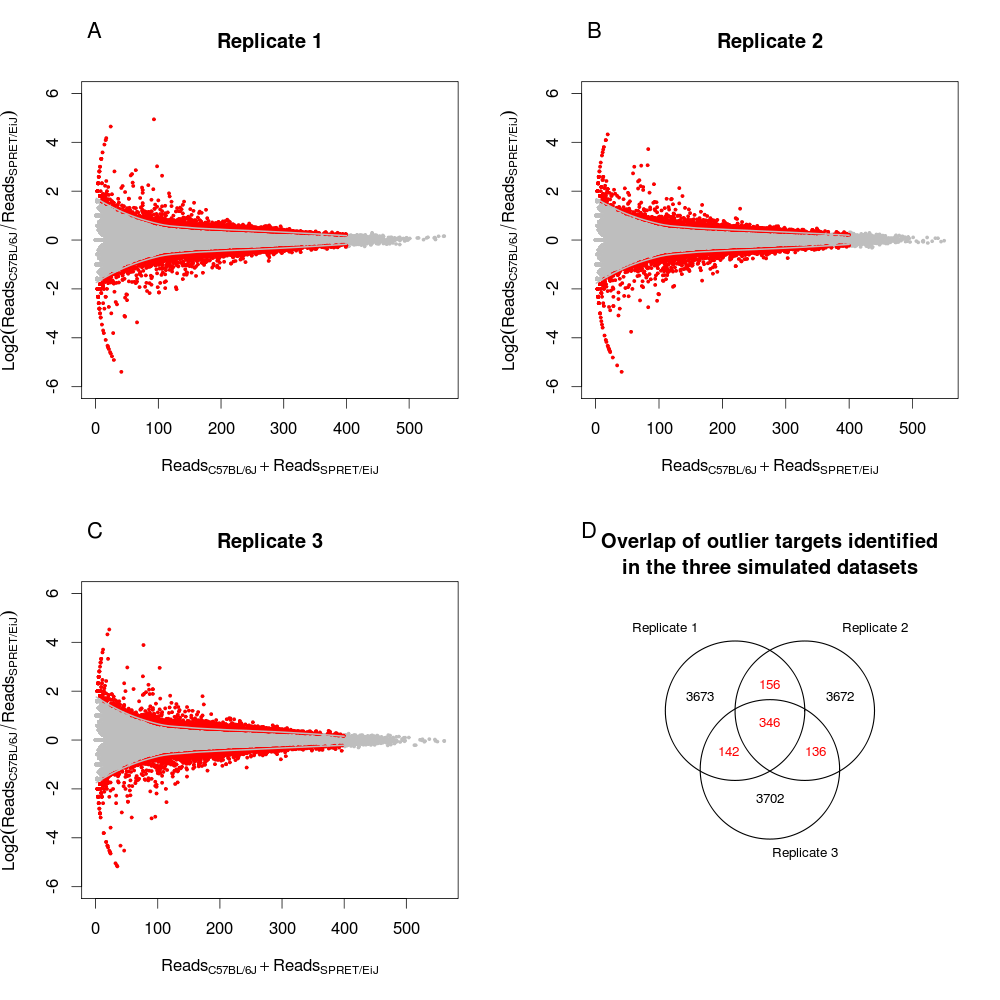

Supplement: Additional file 1 — Exclusion of targets with other complicating factors based on simulated data. (A-C) MA plots comparing the number of sequencing reads that overlapped with a target derived from C57BL/6 J with that from SPRET/EiJ allele based on three simulated exome sequencing data. The sum of the number of sequencing reads that could be mapped only to either genome was plotted on the X axis, and the log2 transformed ratio between the number of reads derived from C57BL/6 J and that from SPRET/EiJ allele was on the Y axis. The red dots represent outlier targets, i.e. their log2 ratio falling outside the 0.5% ~ 99.5% quantiles. (D) The overlap of outlier targets identified in the three replicate simulations. The 780 targets identified as outliers in at least two replicates (shown in red) were discarded in subsequent analysis. [file 1471-2164-14-492-S1.png]

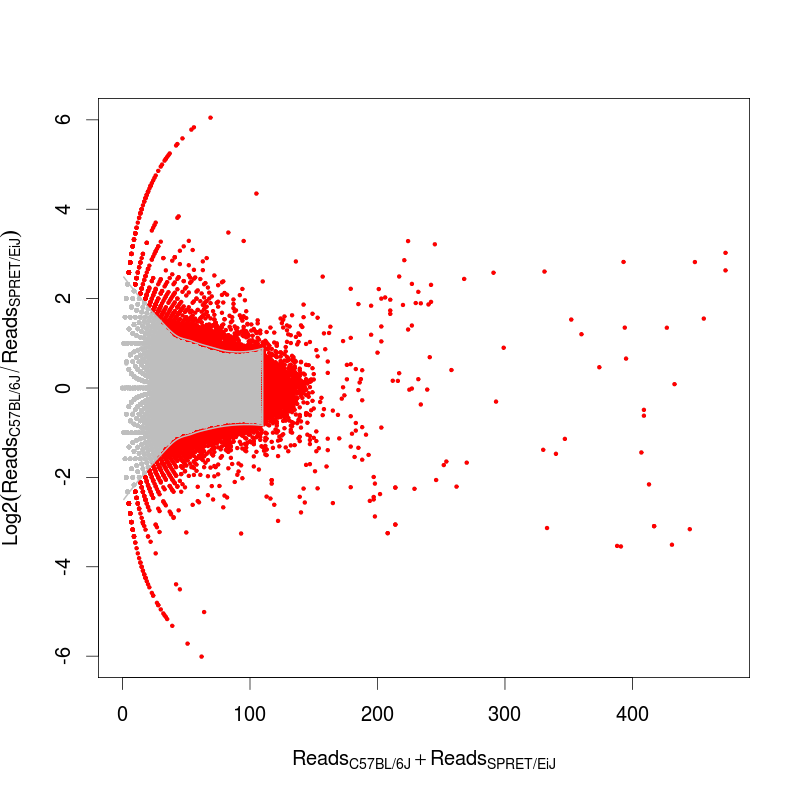

Supplement: Additional file 2 — Exclusion of targets with other complicating factors based on WGS data. MA plot comparing the number of sequencing reads that overlapped with a target derived from C57BL/6 J with that from SPRET/EiJ allele based on WGS data. The sum of the number of sequencing reads that could be mapped only to either genome was plotted on the X axis, and the log2 transformed ratio between the number of reads derived from C57BL/6 J and that from SPRET/EiJ allele was on the Y axis. The red dots represent outlier targets, i.e. their log2 ratio falling outside the 0.5% ~ 99.5% quantiles or the sum of the number of sequencing reads being more than 110. All the 7525 outliers were discarded in subsequent analysis. [file 1471-2164-14-492-S2.png]

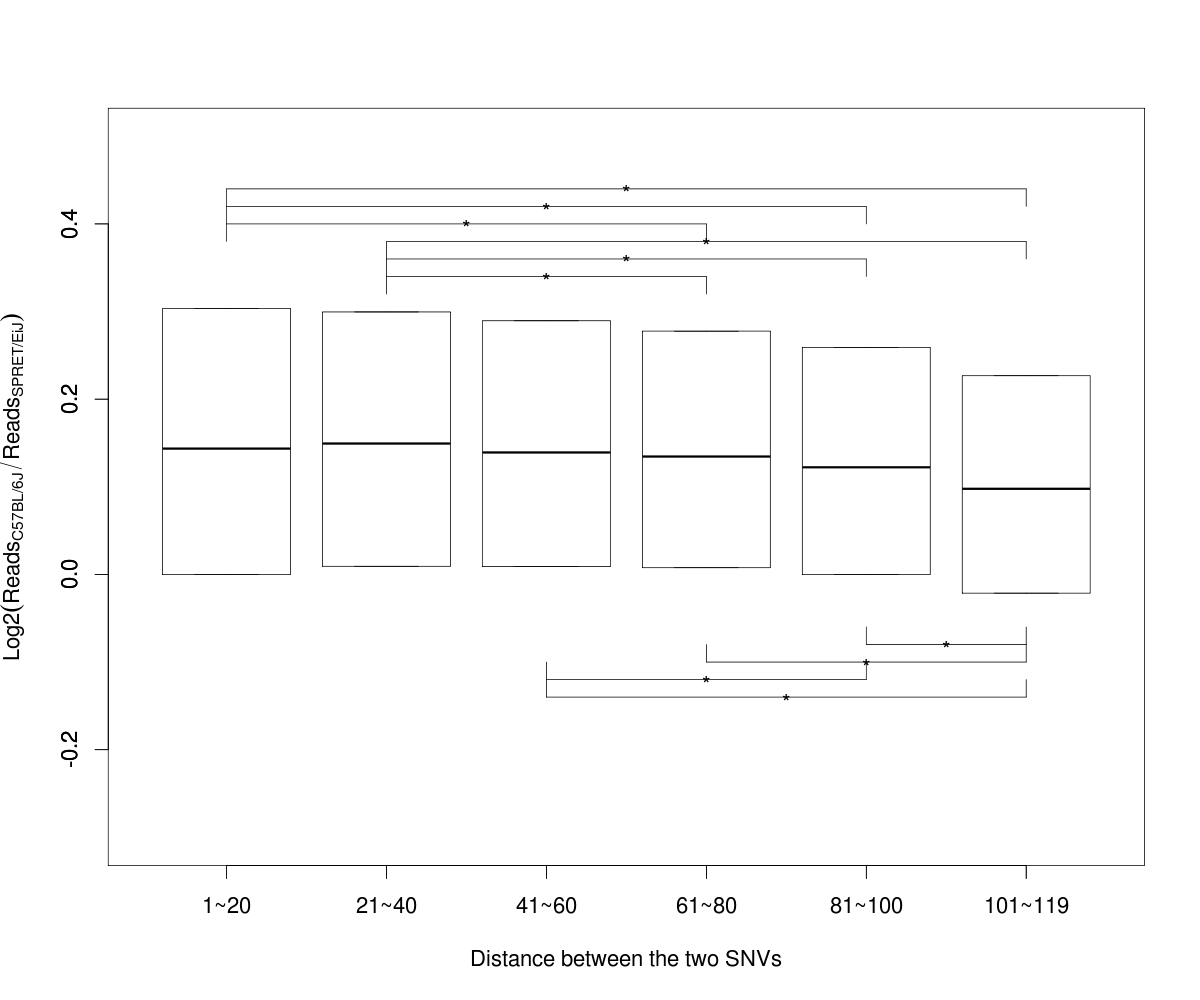

Supplement: Additional file 3 — Impact of distribution of multiple SNVs in the target regions on the capture bias. To check whether the SNVs distribution within the target regions could affect the capture efficiency, we focused on the 64,470 targets containing two SNVs, but no indels and grouped them by the distance between the two SNVs. The distance between the two SNVs was shown on the X axis, and the log2 transformed ratio between the number of reads derived from C57BL/6 J and that from SPRET/EiJ allele was on the Y axis. Compared with the targets with two SNVs being far away, those with the two SNVs being right next to each other displayed higher biases in the capture efficiency. * denotes statistically significant differences. [file 1471-2164-14-492-S3.png]
